# Ferroptosis by Lipid Peroxidation: The Tip of the Iceberg?

**DOI:** 10.3389/fcell.2021.646890

**Published:** 2021-03-25

**Authors:** Xin Chen, Rui Kang, Daolin Tang

**Affiliations:** ^1^Guangzhou Municipal and Guangdong Provincial Key Laboratory of Protein Modification and Degradation, The Third Affiliated Hospital, School of Basic Medical Sciences, Guangzhou Medical University, Guangzhou, China; ^2^Affiliated Cancer Hospital & Institute of Guangzhou Medical University, Guangzhou, China; ^3^Department of Surgery, UT Southwestern Medical Center, Dallas, TX, United States

**Keywords:** ferroptosis, lipid peroxidatiion, POR, CYB5R1, hydrogen peroxide

Ferroptosis is a type of regulated necrosis mainly caused by iron-mediated lipid peroxidation (Tang and Kroemer, 2020). Ferroptosis dysfunction is implicated in various diseases (Stockwell et al., 2017, 2020; Tang et al., 2020), especially cancer (Chen et al., 2021). Lipid peroxidation is a biochemical process in which free radicals (e.g., hydrogen peroxide [H_2_O_2_], superoxide [${\text{O}}_{2}{•}^{-}$], and the hydroxyl radical [•OH]) attack lipids containing carbon-carbon double bonds, especially the polyunsaturated fatty acids (PUFAs) of the plasma membrane or membrane-enclosed organelles (Ayala et al., 2014). Early studies have shown that the production of toxic phospholipid hydroperoxide (PLOOH) during ferroptosis requires the activation of acyl-CoA synthetase long-chain family member 4 (ACSL4)-lysophosphatidylcholine acyltransferase 3 (LPCAT3)-lipoxygenase (ALOX) axis (Yuan et al., 2016; Doll et al., 2017; Kagan et al., 2017). However, despite this significant pathway, the process of lipid peroxidation, especially the biological source of free radicals in ferroptosis, remains poorly understood (Zou et al., 2020a). Complementing previous findings that the oxidoreductases cytochrome P450 oxidoreductase (POR) is a positive regulator of ferroptosis (Zou et al., 2020b), a recent study by Yan and colleagues demonstrated that coupling of POR to cytochrome B5 reductase 1 (CYB5R1) mediates the production of H_2_O_2_, which drives lipid peroxidation and subsequent ferroptosis through the iron-catalyzed Fenton reaction (Yan et al., 2020) (Figure 1).

**Figure 1:**
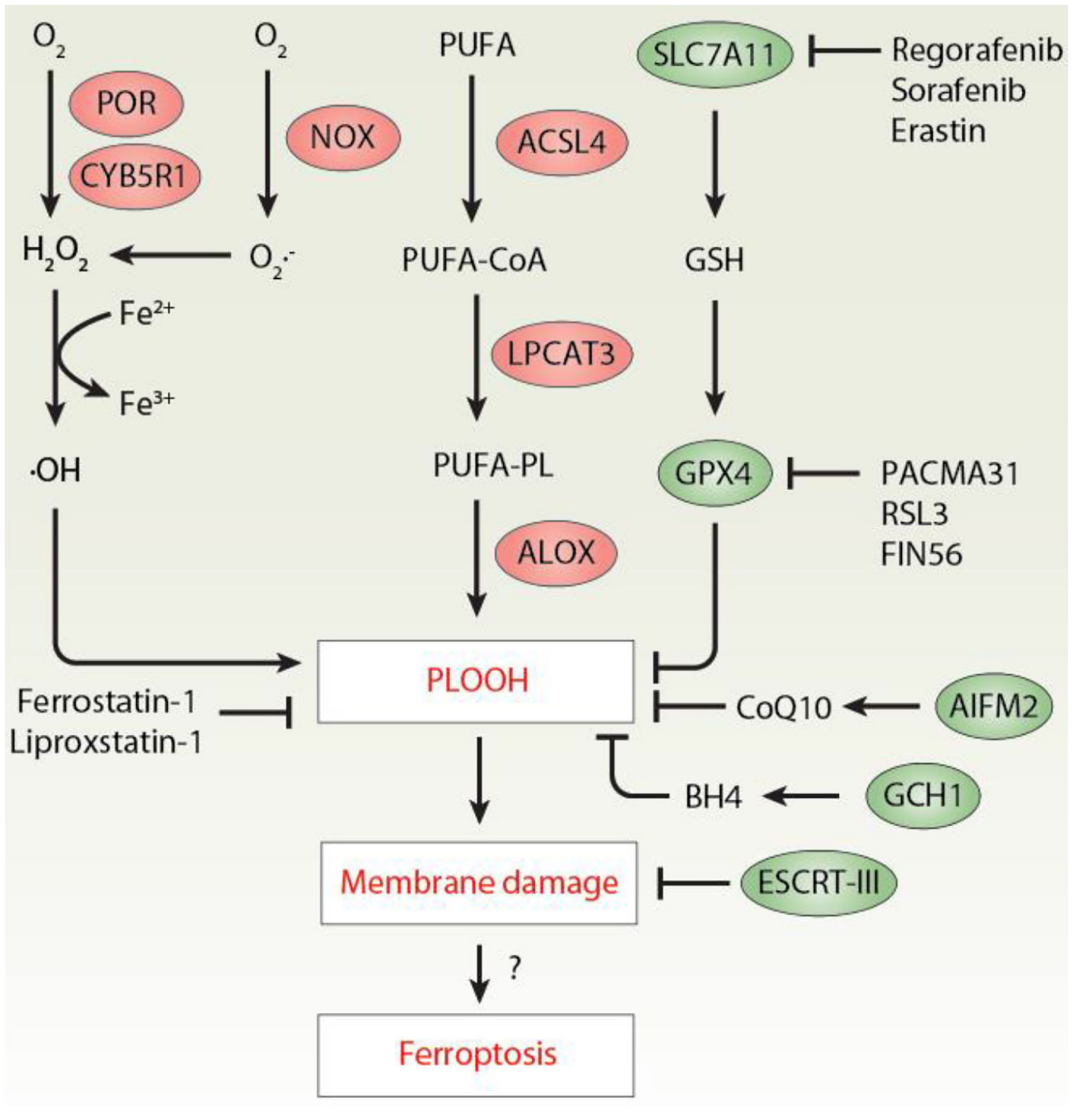
The mechanism of lipid peroxidation in ferroptosis. Excessive lipid peroxidation plays an important role in promoting ferroptosis by producing toxic PLOOH. At least three pathways mediated by POR/CYB5R1, NOX, and ALOX contribute to PLOOH production. In contrast, activation of the SLC7A11-GSH-GPX4, AIFM2-CoQ10, GCH1-BH4 axis limits lipid peroxidation during ferroptosis. In addition, PLOOH-mediated membrane damage can be repaired by the ESCRT-III membrane scission complex. ACSL4, acyl-CoA synthetase long-chain family member 4; AIFM2/FSP1, apoptosis inducing factor mitochondria associated 2; BH4, tetrahydrobiopterin; CoQ10, coenzyme Q10; CYB5R1, cytochrome B5 reductase 1; ESCRT-III, endosomal sorting complex required for transport-III; GCH1, GTP cyclohydrolase 1; GPX4, glutathione peroxidase 4; GSH, glutathione; H_2_O_2_, hydrogen peroxide; PLOOH, phospholipid hydroperoxide; POR, cytochrome P450 oxidoreductase; PUFA, polyunsaturated fatty acids; LPCAT3, lysophosphatidylcholine acyltransferase 3; ALOX, lipoxygenase; NOX, NADPH oxidases; PUFA-PL, polyunsaturated phospholipids; SLC7A11, solute carrier family 7 member 11.

First, the authors combined cell viability measurement with liquid chromatography-tandem mass spectrometry (LC-MS/MS) analysis and found that the small molecule PACMA31 is a novel ferroptosis activator by inhibiting glutathione peroxidase 4 (GPX4), instead of its well-known target protein disulfide isomerase (PDI). GPX4 is a phospholipid hydroperoxidase, which uses glutathione (GSH) to reduce PLOOH to lipid alcohols during ferroptosis (Yang et al., 2014). The production of intracellular GSH is typically initiated by the amino acid antiporter system xc^−^, consisting of solute carrier family 7 member 11 (SLC7A11) and solute carrier family 3 member 2 (SLC3A2). In addition to the classical SLC7A11 inhibitors (e.g., erastin and sorafenib), subsequent drug screening also found that regorafenib can inhibit SLC7A11, thereby enhancing the anticancer activity of PACMA31 in OVCAR-8 (human ovarian cancer cell line) cells *in vitro* and *in vivo*. The synergistic effects of PACMA31 and regorafenib (hereinafter referred to as P/R) also indicate that SLC7A11 and GPX4 have non-overlapping functions in ferroptosis.

Second, the authors performed unbiased genome-wide CRISPR-Cas9 genetic screens in HeLa (human cervical cancer cell line) and OVCAR-8 cells and found that ACSL4 and POR are top candidate genes responsible for P/R-induced ferroptosis. Functional and lipidomics assays confirmed that POR-depleted cells partially resist ferroptosis and are accompanied by a decrease in oxidized phospholipids in response to P/R or classical ferroptosis inducers (e.g., erastin, RSL3, and FIN56). The authors further conducted a small-scale RNAi screening of several oxidoreductases and found that CYB5R1 has a significant promotion effect on POR-related ferroptosis. In contrast, other downstream partners of POR, such as cytochrome P450 family proteins (CYPs), cytochrome B5 type A (CYB5A), squalene epoxidase (SQLE), and heme oxygenase 1 (HMOX1/HO1), were not associated with POR-mediated ferroptosis. These findings emphasize the pro-ferroptotic synergy between POR and CYB5R1 (Yan et al., 2020), although early studies have shown that POR alone is sufficient to promote ferroptosis (Zou et al., 2020b).

Third, the authors explored the mechanism of action of POR and CYB5R1 in regulating lipid peroxidation during ferroptosis. They proved that the electron transport function of POR/CYB5R1 is necessary for ferroptosis induction due to the production of H_2_O_2_. Consequently, exogenous H_2_O_2_ restored the sensitivity of POR-depleted cells to various ferroptosis inducers. In contrast, the overexpression of catalase (CAT), an enzyme responsible for the degradation of H_2_O_2_ to water, prevented ferroptosis. It is clear that iron and H_2_O_2_ can cause biological damage through the Fenton reaction, thereby generating •OH and higher oxidation states of the iron (Fe^3+^) (Ayala et al., 2014). As expected, POR/CYB5R1-mediated H_2_O_2_ production further triggered the iron-dependent Fenton reaction and subsequently malondialdehyde (MDA, a byproduct of lipid peroxidation) production. Importantly, liposome leakage measurement and transmission electron microscopy observations confirmed a direct role of POR/CYB5R1-dependent lipid peroxidation in promoting membrane rupture in a cell free system. This process of membrane oxidative damage was reversed by ferroptosis inhibitors (e.g., ferrostatin-1 and liproxstatin-1).

Finally, the authors assessed the significance of POR-dependent ferroptosis in autoimmune hepatitis induced by concanavalin A in a mouse model. Compared with the control group, treatment with POR shRNA *via* the adeno-associated virus (AAV) attenuated concanavalin A-induced acute liver injury and death, which was related to the reduction of MDA and prostaglandin-endoperoxide synthase 2 [PTGS2, a marker of ferroptosis *in vivo* (Yang et al., 2014)] levels in liver. Thus, POR-dependent ferroptosis may be implicated in concanavalin A-induced experimental hepatitis. It remains to know whether the depletion of POR regulates concanavalin A-induced H_2_O_2_ production and ferroptosis *in vitro*.

In summary, the current research seems to reveal a tip of the iceberg among the intricate interactions between POR/CYB5R1 and ferroptosis (Yan et al., 2020). Although these findings extend our understanding of the mechanisms leading to the activation of lipid peroxidation (Chen et al., 2020), they also raise additional intriguing questions regarding the process of ferroptosis. For example, H_2_O_2_ is currently the most widely used apoptosis or necrosis inducer because of its extensive cytotoxic effect on almost all cell types (Saito et al., 2006). This study also found that other transition metal ions (except iron) do not mediate POR/CYB5R1 to catalyze the formation of MDA (Yan et al., 2020). However, how does POR/CYB5R1 selectively mediate the iron-dependent Fenton reaction and H_2_O_2_-related ferroptosis? In addition, there are many unresolved questions in this study by Yan et al. What is the relationship between POR/CYB5R1 and other lipid peroxidation pathways, such as ALOXs (Yang et al., 2016; Wenzel et al., 2017; Chu et al., 2019; Li et al., 2020)? How does the lipid peroxidation mediated by POR and CYB5R1 cause cell membrane damage? Are there any pore forming proteins that mediate ferroptosis induced by POR/CYB5R1 activation? Recent studies have shown that the endosomal sorting complex required for transport-III (ESCRT-III) machinery is involved in repairing damaged plasma membranes caused by various stimuli (Liu et al., 2020), including ferroptosis inducers (Dai et al., 2020; Pedrera et al., 2020). Does activating POR/CYB5R1 signal also trigger the ESCRT-III membrane repair pathway? Despite this complexity, further elucidation of the feedback loop of oxidative damage and antioxidant defense will reveal novel treatments that can benefit patients with unrestricted lipid peroxidation (Kuang et al., 2020).

## Author Contributions

XC, RK, and DT conceived of the topic for this opinion. All authors listed have made a substantial, direct and intellectual contribution to the work, and approved it for publication.

## Conflict of Interest

The authors declare that the research was conducted in the absence of any commercial or financial relationships that could be construed as a potential conflict of interest.
